# Surgery and Anatomy

**Published:** 1877-07

**Authors:** Jno. E. Owens


### SECTION III. SURGERY AND ANATOMY.

Reported by JNO. E. OWENS, M. D.

TUESDAY JUNE 5—FIRST DAY.

This section was called to order at 3 o'clock p. m., and organized by the election of Dr. F. H. Hamilton, of New York, chairman, and Dr. J. E. Owens, of Chicago, Secretary.

The first paper presented for the consideration of the section, was entitled,

### THE VALUE OF EXTENSION IN THE TREATMENT OF FRACTURES OF THE FEMUR.

It was read by the author, Dr. J. T. Hodgen, of St. Louis, Mo.

The paper was looked upon by the section as a criticism adverse to the treatment of the above-mentioned fracture by fixed dressings, namely, by plaster of Paris, etc. Dr. Hodgen, in his argument, simply expressed the thought, that no fracture of the thigh can be properly treated without more extension than can be secured by fixed dressings. His paper thus concludes:

1. Continued extension of the femur is essential to the best results.

2. This is not to be secured by lateral supports of *any* kind.

3. This can be secured by suspending the limb.

4. Suspension furnishes the best means of allowing motion to all parts of the body.

"For this reason," says Dr. Hodgen, "that some prominent members of the profession have given utterance to language which has been misunderstood—which has been supposed to express the thought that every fracture of the thigh could be so treated by plaster of Paris dressing as to prevent shortening, I am especially anxious that the profession shall be set right in this matter, lest we be brought to bear testimony against ourselves in courts of justice." He then brought to the notice of the section the changes that occur in a fractured limb, and in the perineal band, during the use of the long splint of Liston, namely, the almost daily elongation of the band, the progressive atrophy, from pressure of the tissues about the perineum, the prominence of the rounded thigh, the fullness of the outer part of the leg; and claimed that under these circumstances,—and that, too, in spite of the proper management of the above-mentioned splint,—the limb shortens by muscular contractility.

The author's objections to the plaster of Paris dressing, as used by Sayre and others, for fractured femur, are the following:—"Atrophy will occur rapidly and unequally; the muscular tissue will constantly tend to produce the shortening which the atrophy at the points of pressure of the extending and counter-extending forces will permit." "After a week you can put the finger between the foot and the plaster-case, while one may place a hand between the plaster and the hip." The plaster-case in front upon percussion will yield a resonant sound. Atrophy of the limb has supervened, and the plaster being no longer a fixed dressing, readjustment becomes necessary. In the meantime, the limb has shortened within the plaster-case. Dr. Hodgen likewise took issue with those surgeons who maintained that this shrinkage will not be allowed to occur;—who prevent it by permitting the patient to get up, to move about the room, to go out to attend to business. After quoting from Dr. Sayre's report to the Surgical Section of the Am. Medical Association, 1874, to the effect that it is "better to keep the patient up and walking about occasionally, as the limb will then fill with blood and retain its accuracy of fit to the plaster-casing" etc., etc., Dr. Hodgen acknowledges that it is quite easy to "understand that erect posture would allow an increased quantity of blood to flow into a pendant limb; that the returning flow would be retarded, and that the plaster-case would be fitted by the œdematous limb;" but he deems it absurd to suppose that the tissues at the points where extension and counter-extension are made, are kept so tightly filled with blood and serum as to make adequate extension on the limb. It is admitted that the weight of the limb as an extending force would be sufficient, were it possible to keep the patient erect during the time required for the accomplishment of firm union. Oblique suspension, he continues, by means of Nathan R. Smith's anterior splint, or modification of it, is the only possible way in which unvarying extension can be applied. The discussion upon this paper and upon other methods of treatment, than the one advocated by Dr. Hodgen, continued till the hour for adjournment. The Section adopted the following resolution, offered by Dr. W. F. Peck, of Iowa:—

"*Resolved*, That it is the opinion of this Section, that shortening, in cases of fracture of long bones is the rule in practice, regardless of any of the plans of treatment now in use."

(The paper was referred to the publication committee.)

WEDNESDAY, JUNE 6—SECOND DAY.

ORGANIC STRICTURE OF THE URETHRA FROM MASTURBATION,

WITH A BRIEF ACCOUNT OF ITS PATHOLOGICAL SIGNIFICANCE. By SAMUEL W. GROSS, A. M., M. D. Dr. Gross just directed attention to a report in the Medical and Surgical Reports, May 5, 1877, of fifteen cases of urethral stricture from masturbation, complicated by morbid sensibility of the canal, and attended with more or less marked disturbance of the generative functions. Since that time, three additional examples have come under his notice in private practice. In these, there existed sexual debility, as indicated by premature and painful ejaculation, or by difficulty in commanding erection. He met with still another case in Philadelphia, affording a total of 19 examples out of 138, from all causes. He was convinced by this unusually large proportion, 1 to 7¼, of strictures from onanism, that the habit was a far more frequent source of urethral coarctations, than has been heretofore supposed. In only two of his cases was the stricture of so small a calibre as to give rise to difficulty of urinating. In all of the remaining instances, it was detected in persons who consulted him on account of sexual trouble, irritability of the bladder, gleety prostatic or involuntary discharges, chronic enlargement of the epididymis or irritable testis. From carefully conducted investigations, Dr. Gross maintains that masturbation may produce as intense an urethritis as gonorrhœa, at all events that protracted chiromania is sure to give rise to subacute or chronic inflammation of the urethra, which will eventuate in the formation of stricture just as frequently as does gonorrhœal inflammation; that organic stricture may be found as often in the subjects of chronic onanism as in the subjects of chronic clap.

It is held by most writers, including Sir Henry Thompson, that stricture of the urethra is never traced to masturbation.

In the report above referred to, the author endeavors to explain that this oversight was due to the fact that, out of France, surgeons rely more upon the ordinary elastic bougie, metallic sound, or catheter, for examining the urethra, than upon the soft exploratory bougie of Leroy, with which a stricture cannot elude detection. The urethral stricture, resulting from masturbation, presents certain peculiarities when compared with coarctations from other causes, namely, it is usually solitary, deeply-seated, and not irritable, although it is almost invariably associated with prostatic hyperæsthesia. Of the 19 cases from onanism, on the other hand, all were solitary except three, and in one there was another stricture near the external orifice. The most common situation of the coarctation was 5 7-10 in. from the meatus, or in the region of the bulbo-membranous junction, and as regards the degree of coarctation, it may be accepted, as a rule, that the stricture will be found to be above the medium size, or above number 15 of the French catheter scale, since, in only two instances, representing three strictures, was the calibre below that number, being repeatedly 13, 14 and 14. The average was 17.

The forms of the disease generally met with, are the linear and annular. The indurated annular is rare. In several instances in which the treatment by dilatation extended over a period of several months, the bulbous explorer defined the band, as clearly as in the first instance. The strictures of onanists are not irritible, as they evince no proneness to disturbances of the nervous system, and rarely bleed on instrumental contact; except in one case, there was morbid sensibility of the prostatic portion of the urethra.

Dr. Gross has tested the accuracy of his views upon this matter, by his investigations among the insane of the Philadelphia Hospital, and the Pennsylvania Hospital for the Insane. In some of the insane, the question of gonorrhea could not he entertained because the subjects were admitted at too early an age, and had never left the hospital. In the fifteen examples in the paper previously referred to, those who underwent treatment and faithfully observed instructions, seven were cured by general measures and internal urethrotomy; one was not benefited, the case being that of a hypochondriac, while one was improved merely by the use of steel bougies of gradually increasing sizes. In one case a most distressing neuralgia of the testis was at the same time relieved, while in several, irritability of the bladder, and gleety and prostatic discharges ceased. In one instance only was there true spermatorrhea.

Owing to the frequency with which spermatorrhea follows onanism, the author is led to believe that stricture will be found to be an essential feature in its pathology. "The mere fact of the discovery of urethral lesions in every masturbator confined in an asylum for the insane, should arouse the attention of the superintendents of such institutions to those lesions, as important elements to be considered in the treatment of patients whose mental condition is ascribed to onanism. The most fruitful sources of the general paralysis of the insane are sexual excesses and masturbation. If the removal of the exciting cause in the curious case of reflex paraplegia, from congenital phimosis and adherent prepuce, first observed by Dr. Sayre, results in recovery, may it not be possible in general paresis to prevent functional states from passing into organic disease? Dr. Gross is convinced by an examination of four cases of general paresis, that there is a source of reflex irritability in the urethra.

I regret that I am unable to give a full abstract of Dr. W. T. Briggs' paper on

MEDIO-BILATERAL LITHOTOMY.

It was taken by its author to be re-written, and hence I can only give a portion of the discussion upon the subject. Dr. Briggs, in his paper, in the first place desired to bring to the notice of surgeons this operation, the advantages of which, he feared, were under-valued, and then took the ground that stone in the bladder was most conveniently and safely dealt with by the employment of this operation.

Dr. Gouley, of New York, expressed admiration for Dr. Briggs' enthusiasm for Civiale's operation of medio-bilateral lithotomy, but was opposed to making any operation for stone an exclusive one. The operation should be selected for the case, and not the case for the operation. Lithotomy in any of its methods should not be the exclusive operation, nor should lithotrity. They are perfectly distinct from each other. Where one is indicated, the other is contra indicated. The skill of the surgeon lies in determining which of the two operations should be done, for it is wrong to do lithotomy where lithotrity can be done. I do not believe, as many now say, that the operation of lithotrity is practicable only in very small calculi, nor would I say that it was positively prohibited in large calculi. The circumstances to be taken into consideration are the condition of the urethra, its degree of permeability, its degree of sensitiveness, the condition of the bladder and mucous membrane, the condition of the kidney, the general condition of the patient. In the case of a man with a very irritable bladder, seeking relief every moment of his life, the bladder, perhaps, contracted over a small deposit, the operation of lithotrity would only make him worse.

A well performed cystotomy, a good free incision of the neck of the bladder in such a case, would not only give relief to the sufferer, and afford him drainage for his bladder, but would eventually cure his cystitis. Take the other extreme. A man of 68 or 70, who is walking about, is rather lame and heavy, can take very little exercise without considerable discomfort ; he may not be disturbed for urination more than once in a couple of hours, but he feels that there is something wrong. There are disagreeable sensations about the rectum and bladder. He has been examined repeatedly, perhaps, and nothing has been found. Occasionally there is a little hemorrhage. He thus goes on for several years till some one discovers a stone of considerable size in his bladder. The surgeon finds to his surprise that he is able to seize the stone and move it about in the bladder; he thinks of the enlarged prostate, and believes that cutting might prove dangerous, because of the great vascularity of the parts, the large size of the stone, and besides there is some median enlargement. The question of lithotrity comes up, and it is finally decided upon. The surgeon grasps the stone; it is moderately friable; he breaks it up and is unable to remove the fragments, perhaps, by the natural way. The bladder does not empty itself, so he makes use of the evacuating catheter. He has six or seven sittings. There is no chill, no fever, no unpleasant symptoms, and his patient gets well. There is no *best* method. The best method is that which promises the greatest success to the individual case. We must study—study each case as if we had never seen anything like it before. It is injudicious to operate at once. It is often injudicious to examine at once. Get acquainted with the patient, coax him and coax his bladder, and see how it and he behave, and then all will go perfectly smooth. My second case of lithotrity, (said the speaker) a tall, lank man, who had suffered from stone for seven or eight years, could not be touched; he either had chills, or sickness in one way or another. It was intended at first to cut the patient, but the urethra became more tolerant. A whole month was consumed in preparatory treatment. He became so docile under the proper treatment, that lithotrity was determined upon, and at the end of twenty-four sittings the patient had been relieved of an ounce and a quarter detritus. There were two stones. Lithotrity will only be employed when surgeons make up their minds to exercise more patience, to learn better how to deal with a patient. For the sake of comparison we want the publication of unsuccessful cases. The speaker begged every gentleman who has cases of stone, and especially if they be unsuccessful, to publish them. We want all the cases, in order that we may place a just estimate on the operation or the various methods of operating. Dr. Gouley's own mortality is one in eighteen cases in lithotrity. When lithotomy is indicated, that is when there is inordinate cystitis, inordinate vascular irritability, no operation in which the neck of the bladder is not divided will prove quite satisfactory. It may relieve, but it will not prove quite satisfactory.

Treatment of Fractured Ribs by Extension and Expansion of the Thorax and Retention by Plaster of Paris Bandage. By Dr. Lewis H. Sayre, N. Y.—In January last Dr. Sayre was requested by Dr. Sims, of New York, to see an old lady, 78 years of age, very greatly emaciated, who had been an invalid for a great many years, having, in early life, had inflammation of the left lung, resulting from abscess, leaving her collapsed in the left side, the ribs on the left side completely overlapping, so as to entirely include the intercostal spaces. This lady, about eight days previously, had fallen in the hall on her left side against the corner of a marble slab, breaking the second, third and fourth ribs, about the middle. She had great difficulty in breathing, and almost incessant cough, and she was almost unable to sit down. Drs. Sims and Austin Flint attended her. The only way in which she could be kept in a comfortable position was by sitting upon the edge of a sofa between two assistants, and her arms reaching over their shoulders, her body being bent forward and her head resting upon a pillow upon a table. She had been in this position six days before Dr. Sayre was called to see her. Her medical attendants had tried all the ordinary methods for the treatment of fractured ribs, without giving relief. Watching the attitude of the old lady with her arms stretched tightly over the shoulders of her assistants, and learning that whenever a change of assistant was necessary to relieve those already on duty from fatigue, the pain was almost unendurable, and the only time she could get easy again was when she got in the position I have described, I discovered that it was simply by the extension of her arms, and the consequent distention of the pectoral muscles and other thoracic muscles that the ribs were held apart, as far as possible, and she was comparatively easy. After making traction on her hands and pulling her up as far as possible, spinal curvation with anchyloses rendering it impossible to straighten her, she was instructed to take a deep inspiration, and then she said that she felt more easy than she had at any time since the accident. Having made her undershirt fit as smoothly as possible, the plaster of Paris bandages were applied which Dr. Sayre uses for Pott's disease. The arms were held in the manner described until the plaster became hard, after which the old lady got into a chair in a comfortable position, and began a pleasant conversatio. During the seven or eight days previous to the application of the plaster jacket, it was almost impossible to get breath enough to articulate a few sentences, and her cough was almost incessant.

From the moment her arms were extended there was not a single cough. The patient died of exhaustion in nine or ten days. The comfort that this lady experienced after the application of the jacket, compared with the agony that existed before its use, was a matter for consideration. It occurred to Dr. Sayre that it was the position in which she had instinctively placed herself that had given her comfort, and that the fixation of the trunk by the immovable apparatus was the plan of treatment. Dr. Sayre thought at the time that the method was original with him, but Dr. St. John, of New York, informed him that he had already applied plaster of Paris in the same way, when he was house-surgeon at Bellevue Hospital.

Dr. Sayre has applied the jacket in two other cases of fractured ribs by setting the patient in a chair or stool without a back to it, and having two assistants, one on either side, hold a broomstick over their heads, so that the patient can extend the arms over the broomstick, and at the same time take a deep inspiration. The ribs become thus accurately adjusted. The instant the parts are in accurate apposition, and are held by the muscles, the patient will let you know it, either by his face or his words; then apply the bandage and instruct the patient to maintain the position till the plaster soldifies.

Dr. I. N. Quimby, of New Jersey, then read a paper entitled

OPERATIONS ON PARALLEL BONES WITH LOSS OF SUBSTANCE, of which the following is a brief abstract: Having been called to attend a patient suffering from a compound comminuted fracture of the tibia and fibula, a little below the middle, he ascertained that there was a complete loss of 1¾ inches of the tibial shaft and corresponding destruction of the soft parts.

Dr. Quimby then proceeded to perform the following operation, the patient having been anæsthetized. An assistant seized the limb above and below the seat of injury, and bent the member at the wound, forming nearly a right angle, exposing to view as much as possible the ends of the fragments. Finding the remaining upper and lower fragments of the tibia sharp, jagged and irregular, the wound was enlarged, the soft parts dissected away from the broken ends of the tibia just sufficient to apply a small metacarpal saw, and these extremities removed. There was left a tibial deficiency one inch and three-fourths in length.

The operator then carefully "worked his way" through the wound to the lower fragment of the fibula, dissecting the soft parts away from the end of the bone, and seized it when exposed with a pair of bone forceps, which were firmly held by an assistant while Dr. Quimby sawed off one and three-fourths of an inch of the fibula. The parts were then placed in accurate apposition, care being taken to avoid the interposition of the soft parts between the osseous extremities. The limb was then placed in an ordinary fracture-box, with lateral hinged doors and bran stuffing. To secure immobility, a large piece of adhesive plaster was made to cover the entire sole of the foot, and was then reversed over, and fastened to, the foot-board, being afterward further secured with circular strips and a roller bandage. A compress was also fastened over the lower anterior part of the leg as near the wound as possible, which was held in place by a strip of plaster passing across the top of the box and secured through perforations in the latter. Thick, broad compresses were similarly applied above and below the knee, and also on each side of the limb.

Immobility was thus maintained for seven weeks, the wound being meantime covered with a lotion of carbolic acid. A profuse purulent discharge ensued, which gradually diminished, and at the end of eight or nine weeks union had occurred. The limb was then removed from the box, a plaster of Paris bandage applied with proper fenestrum, and retained for three weeks. In three months the patient was walking with a cane. (A photograph was exhibited, showing the patient supporting his weight upon the two limbs, the wound being cicatrized.) The patient has a slight limp in his gait, and the shortening corresponds to the length of the segment removed, $1\frac{3}{4}$ of an inch. The patient had been seen by Drs. Post, Sayre, Crosby and others.

Dr. Quimby concluded:

1. That other limbs might be saved by such procedure.

2. That the attempt should be made in the case of parallel limbs similarly injured.

3. That the operation was original with the author.

4. That even *without* fracture of the fibula, a similar procedure would be advisable.

Dr. Bayard, of Quincy, stated that he had enunciated the principle in May, 1874, and in the New York Medical Journal of last year, having secured immobility by drilling holes through the extremities of bones and wiring them together.

Dr. Hodgen, of St. Louis, after inquiring whether every particle of bone and periosteum had been removed by the injury from the 1¾ inch of the tibia found wanting, and being then answered in the affirmative, remarked that there was a danger in reporting such cases lest they might lead to improper practice. The value of periosteum and fragments in such cases was great. In case of any doubt the patient should have the benefit of it.

The Chair remarked that, if there was any doubt respecting the filling up of the space left between the fragments of the tibia, it would, in his opinion, be wiser to fracture if the fibula and permit the broken ends to penetrate the flesh and remain overreaching. This would not diminish the chance of final consolidation, as it not unfrequently occurs from accident.

Dr. Truesdell, of Illinois, concurred in the views expressed by Dr. Hodgen. He reported a case in which he had finally to remove a piece of the tibia, one inch in length and four-fifths of the thickness of the bone. The entire space was filled by granulation, and the cure was complete without shortening or lameness. In some cases he had been able to scrape out the entire shaft of the bone with his finger, for two inches. Periosteum was not necessary to complete repair. The tissue forming the cancellated structure of the bones, is simply an extension of the periosteum, and those cancellated cells produce plastic material convertible into bone.

Dr. Sink, of Indianapolis, also believed in the restoration of bone under such circumstances, believing the source of it to be not the periosteum merely, but the medullary tissue, from which granulations arise even when exposed to the air, if that air be not irritating. Judging from his own experience, and alluding to the observations of Virchow, he believed that the bone in this instance would have formed to fill up the vacant space between the tibial extremities. In one instance he had removed five inches of the tibia, leaving but a little specimen of bone, and reformation occurred, the man recovering with a sound limb.

Dr. Humphrey, of Missouri, concurred in the views already expressed, citing a case treated by himself where three inches and one half of the entire shaft of the tibia were removed, and the periosteum completely destroyed, the fibula being intact. The limb was kept extended upon Dr. Hodgen's splint, after the removal of the fragment, and the result was perfect.

Dr. Quimby referred to a case treated by Prof. Crosby, of Bellevue, where the entire shaft of the tibia was gone for 1½ inches. The patient objected to removal, and only partial restoration occurred; the patient to-day is walking around with a steel splint. In his case, if spicula had been found, he would have attempted restoration. He had himself removed two, three and four inches of bone where the periosteum was partially left, and union had resulted. He believed that cancellated tissue also could reproduce bone.

Dr. Hughes, of Iowa, believed that many present, if called to a similar case, would have performed the operation described. In three instances, the speaker had removed portions of the bones of the fore-arm, and by shortening the limb had obtained excellent results. The operation is therefore not a new one. But where either medullary tissue or periosteum is left, removal, of course, is unjustifiable.

Dr. Hodgen remarked that he now understood one and three-fourths of the tibia was gone in its entire thickness. (Dr. Quimby—"Yes.") In that case the anterior tibial nerve and artery must have been removed with the soft parts, and the danger of mortification being great, amputation should have been performed.

Dr. Clapp, of Iowa, could understand why bone might be removed in the fore-arm, but could not believe it necessary in the case of the fibula.

The Chair reiterated his former opinion, believing the danger of fracturing the fibula and thrusting the ends over each other, to be small. Dr. Quimby went through a very deep wound in his operation—through the inter-osseous space where important vessels lie. He (the Chair) could not sanction the operation.

Dr. Hingston, of Montreal, thought it difficult to reach the fibula, and could not see where the "conservative surgery" came, in the case reported. He could not endorse the practice recommended.

THURSDAY, JUNE 7.—THIRD DAY.

A PAPER ON SUSPENSION AS A MEANS OF TREATMENT IN SPINAL DISTORTIONS. By BENJAMIN LEE, M. D., of Philadelphia, was then read by Dr. Frank Woodbury, of Philadelphia, in the absence of the author.

Three classes of cases were named as those in which suspension might prove useful:

1. Those in which the cervical vertebræ were the seat of disease.

2. Those in which the requisite amount of antero-posterior pressure could not be borne save for a limited time each day (hyperæsthesia, caries, abscess, etc.)

3. Those in which lordosis occurs, or a tendency thereto, in consequence of yielding of the inter-vertebral substance, or partial absorption of the oblique processes.

The author constructed an apparatus called by him "the spinal swing," the principle of which was suggested to him while he was engaged in examining a young patient who endeavored to aid in his spinal extension while in the hands of an assistant. The apparatus consists merely of a pulley fixed above the head of the patient, over which passes a cord which is attached to a chin and occipital strap, the patient himself producing gradually extension by traction upon the cord to which wooden ovals are fastened for greater convenience of handling.

The paper was devoted to the explanation of several illustrative cases, and concluded with the statement that Dr. L. A. Sayre's adoption of this principle in the application of the "plaster jacket," had greatly contributed to the interest taken in the subject by the entire profession, due credit being given the doctor for the introduction of a valuable method of securing both fixation and extension. As to the time when such treatment should be inaugurated, the author urged that delay should not be counselled on account of the feebleness or weakness of a patient, so that a patient affected with spinal disease should be treated as speedily as one suffering from fracture of the extremities.

Dr. Sayre, of New York, did not agree with the sentiment expressed that seif-suspension was useful in spinal caries. All due credit should be given Dr. Lee for the employment of self-suspension in lateral curvature, by far the best treatment yet given to the profession. In self-suspension the hands should always be kept above the forehead, for if lower, the patient might hang himself, therefore an assistant should stand by to prevent accidents. Once suspended fairly, the patient should take two or three deep inspirations to expand the chest.

Now in caries, you require consolidation, and that demands rest, not motion.

Prof. S. D. Gross, of Philadelphia, then read a paper on the "Proximate Cause of Pain."

The following propositions were stated:

1. That the nervous fluid, as it is termed, is precisely similar to, if not identical with, the electric or galvanic fluid, modified, of course, by the play of the vital actions which everywhere exists in the organs and tissues through which the nervous fluid circulates.

2. That the fluid under consideration is generated by the brain, spinal cord and nervous ganglia, and that the nerves are simply passive cords, ropes, or, so to speak, wires for the transmission of the nervous fluid.

3. That what is called pain is due immediately and directly to the obstruction to the transmission of the nervous current, thereby causing an accumulation of nervous fluid at the seat of obstruction.

4. That pain can take place only in connection with a sound state of the brain and spinal cord, or, in other words, that where those organs are seriously affected, there can be no perception whatever of pain or suffering.

5. That pain is modified or influenced by structure, and by the nature of the exciting cause.

Some of them are self-evident propositions; others are to be established only with difficulty.

That what we call pain is due to the obstruction referred to above, is illustrated in the following considerations:

1. "Spasm," as commonly used, designates affections characterized by involuntary muscular contractions, alternating with more or less marked relaxations, often followed by a distinct interval of ease (clonic). Illustrations were given of spasm in tetanus, colic, (bilious and "lead"), stricture of œsophagus, uterine contractions, etc.

2. The second class of cases considered, embraced wounds, the division of tissues in their various relations (contusions, lacerations, etc.,)and the bites and stings of animals and insects. In the simpler cases the pain is slight from temporary obstruction of the nervous fluid, there being a speedy nervous anastomosis, or it may pass off at the seat of the wound instead of accumulating, as in compression or pinch, when the nerve tissue is more or less seriously compressed. In contusions and lacerations and bites of animals, pain is transient from paralysis and non-conduction of the nerve. Stings are painful from toxic causes or serous effusion.

3. Cases where inflammation exists, the process resulting in the production and maintenance of pain, remarkably modified by the structure effected. Whatever may be the nature of the suffering organ, there is always some pain. The more solid the tissue, as a rule, the greater the pain and constitutional disturbance, and obstruction of the nervous current.

4. The same is true of tumors. Pain in scirrhus is severe, in medullary sarcoma insignificant, so in external as contrasted with internal hæmorrhoids.

5. Finally, mere congestion or capillary engorgement may produce the interference described. (*E. g.*, foreign body in the eye, chordee.)

The author further noted the careful protection in the economy of nature, of the nerves passing from center to periphery, and alluded to the pain in lost parts (*e. g.* after amputation), first described by Baron Larrey, when "the spinal cord continues to generate the same kind of nervous fluid which it was in the habit of supplying to the limb, prior to its removal."

The paper on Vaccino-Syphilis, by Dr. B. R. Senseny, of

Pennsylvania, contained a brief reference to the admission by Jenner, that vaccination might introduce into the system a virus other than that intended, and after allusion to the Italian epidemics of syphilis originating in vaccination, concluded with (the main part of the paper) an exposition of the excellent fasciculus of Hutchinson's Illustrations of Clinical Surgery, (vaccination syphilis) which is already in the hands of the profession.

Foreign Bodies in the Ear. By C. J. Blake, M. D., Boston.—Cases in which a foreign body is lodged in the external auditory canal, call perhaps more than other accidents affecting this part of the organ of hearing, for the exercise of a patience and skill, the want of which often entails serious injury to the deeper seated and more delicate parts of the organ of hearing. Voltolini pithily says that the point of danger in the external auditory canal will do much less harm than injudicious attempts at its removal. The later text-books decry any attempts made for the removal of foreign bodies from the ear, that are not made with a full knowledge of the structure and relations of the parts of the ear in question, of the character and location of the foreign body and of the various methods which may be employed for its removal. The paper here contains a brief review of the topography of the external ear. No attempt at removal should be made without proper and sufficient illumination of the parts. The body of the membrana tympani is formed by two layers of fibrous tissue, the fibres of which are so arranged as to give it great strength and elasticity—but not sufficient strength to justify its use as a point of resistance in the attempt at instrumental removal of a foreign body which may be lodged against it.

Cases have come under the author's observation in which for want of an observance of these precautions, the membrana tympani had been ruptured and the malleus torn away. In the majority of cases, even including those in which the foreign body may be firmly impacted, the removal may be effected with little or no pain to the patient, the force used being expended upon the entire surface of the foreign body without using the walls of the canal or the membrana tympani as points of resistance, or indirectly upon the inner surface of the foreign body. Pain as a rule implies injury which might be avoided by patient and delicate manipulation. Slight injury may entail serious consequences to the health of the patient. Since bodies which find their way into the ear vary, the means employed for their removal must therefore vary, and the choice will depend upon the judgment of the surgeon, and the means at his command.

The cases which present the greatest difficulties are those of hard bodies, such as stones, beads, buttons and the like; bodies liable to expand by the absorption of moisture, such as beans, and peas, and impacted masses of epidermis, which resent the action of water and offer no hold for the forceps or hook.

Out of 2,374 cases examined during the past year, in the aural clinic of the Massachusetts Charitable Eye and Ear Infirmary, there were 30 cases of foreign bodies, of this number 14 were cases in which a bean, pea, kernel of corn, or similar substance, had been pushed into the ear. In six cases, insects were removed, and in one case the living larvæ of the common blow fly. In one of the common cases, a bean had remained ten weeks without causing irritation; in another, a small bean had been pushed into the middle ear, in consequence of a previous attempt at its extraction. In the majority of the cases, the foreign body was removed by means of syringing with warm water, which possesses the advantage of permitting the application of the maximum of force with the minumum of danger, the force, moreover, being applied without the foreign body. The object to be accomplished in the use of the syringe, is to establish a body of water between the foreign body and the membrana tympani, connected by a slender column of water between the foreign body and the wall of the canal, with the column of water in the syringe, furnishing, in this manner, the elements of a hydraulic press

Among the most simple procedures, that of Dr. Lowenberg, of Paris, may sometimes prove useful. This consists in the application of a camel's hair pencil, dipped in joiners' glue, to the foreign body, which should first be carefully dried by wiping with absorbing cotton. The glue is allowed to set and the removal the naffected.

Dr. E. H. Clarke, of Boston, employed, in one case, a still more ingenious method for extraction of a smooth glass bead. A thread was passed through a small disc of lead plaster. This was carefully pressed upon the outer surface of the bead, and sunlight concentrated upon it by means of a lens. The plaster softened and adhered, and the bead was easily withdrawn. A loop of wire or horse-hair is often of use in removing a hard body, and by this means possesses the advantage of being but little likely to cause injury by irritation. The common angular forceps, and the blunt hook are more or less useful. The former should never be used beyond the point of sight, nor the latter as a lens, the fulcrum for which is some point on the wall of the canal. The paper concluded with the citation of a few cases which serve to point the moral that in the proper treatment of foreign bodies impacted in the ear, skill, and not force, is required.

A paper on Recent advances in Otology was then read by S. J. Jones, M. D., Chicago.

The advances made in the last two decades, in otology are second only to those in ophthalmology.

Prior to the time of Sir William Wilde but little of practical value was known of the ear and its diseases, and still less of reliable treatment of its affections.

The anatomical division into the external, the middle and the internal ear is important for diagnosis and treatment. The physiological division into the conducting and the perceptive apparatus is important as regards treatment and prognosis.

It seems now well established that the most essential part of hearing is performed by the vestibule; that the cochlea recognizes musical sounds, and that the semi-circular canals preside over the equilibrium of the body, and possibly serve as safety-valves for the other parts of the labyrinth.

The establishment of the fact of the power of accommodation of the ear similar to that possessed by the eye is of recognized value, and may be taken advantage of to develop impaired hearing.

One of the greatest advances is in the elimination of so large a part of those cases once supposed to be nervous deafness, and the establishing of the fact that nervous deafness is rare, and usually supervenes suddenly, and that it is attended, in addition to complete deafness, by nausea, vomiting, unsteadiness of gait in walking, etc. The cases before supposed to be nervous deafness are most frequently found to be the results of defects in the conducting apparatus, instead of in the perceptive, and to be caused by chronic non-suppurative inflammation of those parts.

Inattention in diagnosis may lead to confounding acute inflammation of the labyrinth—especially in children—with cerebro-spinal meningitis.

In the treatment of all affections of the ear, except cases of true nervous deafness, much more can be accomplished than is generally believed by the profession and by the public.

Often the integrity of the membrana tympani may be saved, and even the life of the patient spared, by timely puncture of the membrane, in severe acute inflammation of the middle ear.

Openings in the membrana tympani, whether spontaneously or artificially made, usually heal readily, unless very great loss of substance occur.

Great danger lies in allowing purulent discharge from the ear to continue; never in stopping it, by the removal of the causes which are producing it.

Many cases of deafness are supposed to have been inherited. Deafness is not a disease, but a consequence of disease, or defect, of the organ of hearing, and hence it is difficult to conceive of hereditary deafness.

The subject of deaf-mutism merits more attention than it receives. These cases are more frequently acquired than congenital. The defect is far more frequently in the conducting apparatus than in the perceptive apparatus, and hence more favorable for treatment. Wise and humane as it is to provide shelter and care and instruction for the irremediably deaf and mute persons, it is yet better to seek to remove the cause—where it can be done—which, as its consequence, makes such care and provision needful.

It is with much regret that, for want of space, we merely mention the titles of the papers that were next presented for the consideration of the session. Dr. H. O. Marcy, of Cambridge, Mass., gave an abstract of his paper on plastic splints, and exhibited a plaster mill, used to facilitate the preparation of plaster of Paris bandages.

Dr. Sayre then exhibited a girl, 12 years old. This child can neither talk, walk or feed herself, nor has she direct control of any muscle. She has the appearance of an idiot, says Dr. Sayre, and yet she is not an idiot. The girl has no power to express herself in language at all. The limbs cross in an incoordinate manner. The child has been abandoned as a hopeless case, but in Dr. Sayre's opinion, it is a case of reflex incoordination from genital irritation, and can be cured by the removal of the extremity of the clitoris. (1)

Dr. Edmund Andrews, of Chicago, read a paper entitled, "Studies in rendering incisions painless by means of high velocity," and explained the instrument for making such incisions.

Dr. C. Fayette Taylor, of New York, exhibited a new osteoclast, illustrating, by a case, its use in ameliorating deformity and restoring the power of locomotion, in anchylosis of the hip-joint, with bad position. Abstracts of papers entitled as follows were then read: "The open air treatment of wounds," by J. E. Link; "The relative value of incisions and aspiration in the treatment of empyema," by Dr. H. T. Bowditch; "Elastic bandage in the treatment of varicose ulcers, sprained ankle, enlargement of joints from increase in quantity of synovial fluid, as a result of rheumatism, etc., etc.," by Dr. Henry D. Martin, Boston.

Adjourned sine die.

(1) This case came under my care a few days after adjournment of the Association. I operated, and at the end of a week the result is negative.—J. E. Owens.

